# Functional Trait Divergence Underlies the Spatial Trade-Off Between Water and Nitrogen Use Efficiencies in Northern Tibetan Alpine Grasslands

**DOI:** 10.3390/plants15071076

**Published:** 2026-04-01

**Authors:** Guangshuai Zhao, Mingcong Yan, Peili Shi, Xueying Chen, Huixin Hei

**Affiliations:** 1 Development Research Center, National Forestry and Grassland Administration, Beijing 100714, China; zhaogs.10s@igsnrr.ac.cn; 2 School of Economics and Management, Beijing Forestry University, Beijing 100083, China; yanmingcong@yeah.net; 3 Key Laboratory of Ecosystem Network Observation and Modelling, Institute of Geographic Sciences and Natural Resources Research, Chinese Academy of Sciences, Beijing 100101, China; chenxueying0845@igsnrr.ac.cn (X.C.); heihuixing@126.com (H.H.); 4 College of Resources and Environment, University of Chinese Academy of Sciences, Beijing 100190, China

**Keywords:** alpine grasslands, precipitation gradient, functional trait divergence, resource use efficiency, spatial trade-off, nitrogen limitation

## Abstract

The coupling of water and nitrogen (N) availability critically constrains alpine plant growth and ecosystem productivity, yet the mechanistic links between plant functional traits and resource use efficiencies (rain use efficiency, RUE; nitrogen use efficiency, NUE) along precipitation gradients remain unclear. This study aimed to test whether coordinated shifts in plant functional traits are associated with spatial variation in RUE and NUE across a precipitation gradient on the Changtang Plateau. Here, combining transect surveys with N-addition experiments on the Changtang Plateau, we measured biomass and leaf/root functional traits on four typical grasslands and analyzed the spatial variations in RUE, NUE, and fertilizer use efficiency (FUE). Our results demonstrated contrasting spatial patterns: with increasing precipitation, soil resource availability, community species richness, and biomass significantly improved, and vegetation shifted from a water-conservative strategy in arid regions to a nutrient-efficient strategy in humid regions. FUE increased with precipitation (*p* < 0.05), with low-dose nitrogen addition exerting more pronounced effects in humid regions, indicating greater responsiveness to fertilization. This transition in resource use patterns is underpinned by a coordinated divergence in functional traits: as water limitation eases, communities exhibited decreasing specific root length (high specific root length, SRL) coupled with increasing specific leaf area (high specific leaf area, SLA) along the gradient. Our findings demonstrate that functional trait variation is associated with the optimization of resource acquisition across environmental gradients. These results provide a mechanistic basis for adaptive management in climate-sensitive alpine biomes, where differentiated grassland management schemes may enhance ecosystem productivity—water conservation and reduced disturbance in arid regions, with moderate low-dose nitrogen fertilization and species diversity protection in humid regions. Long-term ecosystem responses to such management approaches require further investigation.

## 1. Introduction

Plants adapt to resource constraints by optimizing their resource use efficiency, yet predicting how vegetation shifts strategies across broad climatic gradients remains a central challenge in global change ecology. As a core indicator of environmental adaptation and survival strategy optimization, the resource use efficiency directly reflects plant resource uptake and transformation capacity [1]. In terrestrial ecosystems, particularly in arid and semi-arid alpine biomes, primary productivity is fundamentally constrained by the co-limitation of water and nitrogen (N) [2]. With climate change intensifying hydrological cycles and altering soil N mineralization rates, the coupling/decoupling of water and nutrient availability has become a critical determinant of ecosystem resilience. Consequently, plant communities must regulate water use efficiency (WUE) and nitrogen use efficiency (NUE) to balance prevailing resource constraints. However, how coupled water and nutrient variations along precipitation gradients shape the spatial patterns of WUE and NUE [3], as well as the underlying mechanisms linking these efficiencies to functional traits, remain poorly understood.

It is crucial to distinguish between precipitation-based resource use efficiency and physiological water use efficiency (WUE). RUE, defined as the ratio of aboveground net primary productivity (ANPP) to mean annual precipitation (g m^−2^ mm^−1^), serves as a community-scale indicator of precipitation use efficiency in grassland ecosystems [4]. This metric, while not equivalent to physiological WUE (which typically refers to the ratio of carbon assimilation to transpiration at the leaf level), provides a practical and easily measurable indicator for large-scale assessment of ecosystem water utilization in contexts where precipitation is the primary water source, hydrological processes are simplified, and vegetation growth is synchronized with precipitation (e.g., Qinghai–Tibetan Plateau grasslands). Physiological WUE, conversely, reflects the internal efficiency of plants in converting water into carbon through photosynthesis [5]. The distinction between these metrics is essential for accurate interpretation of resource utilization patterns across spatial scales.

The coupling of water and nitrogen availability critically constrains alpine plant growth and ecosystem productivity [2]. Precipitation gradients fundamentally influence soil nutrient dynamics through multiple mechanisms, including soil moisture-driven changes in microbial activity and mineralization rates, nitrogen leaching under high precipitation, and the relative availability of organic nitrogen pools [6,7]. As precipitation increases, soil moisture generally enhances microbial activity, accelerating nitrogen mineralization and increasing the availability of inorganic nitrogen for plant uptake [8]. However, in high-precipitation regions, increased leaching may reduce soil nitrogen retention, creating complex spatial patterns of nitrogen availability. This mechanistic understanding of precipitation-driven nitrogen dynamics is essential for explaining the observed resource use patterns across environmental gradients.

The spatial trade-off between WUE and NUE [9,10] is well-established at the individual or leaf scale. For example, N-addition typically boosts carbon acquisition in *Leymus chinensis* to increase WUE but at the cost of reduced NUE, reinforcing a spatial trade-off strategy under co-limited conditions [10,11,12]. However, extrapolating these physiological mechanisms to the community level involves significant uncertainty. Recent studies on *Stipa purpurea* on the Tibetan Plateau have reported synergistic WUE–NUE responses [13,14], suggesting that resource supply dynamics can override physiological constraints [15,16]. Crucially, previous studies have predominantly focused on croplands or individual organs, often overlooking how community assembly processes and interspecific competition modulate resources use strategies. Consequently, it remains unclear whether the spatial trade-off hypothesis holds true across steep precipitation gradients, or if resource heterogeneity drives a shift towards synergistic co-limitation at the ecosystem scale.

Plant functional traits serve as the mechanistic bridges connecting individual physiological strategies to ecosystem functions, specifically, WUE and NUE. These traits fundamentally constrain plants’ abilities to acquire, utilize, and conserve resources. Leaf traits such as specific leaf area (SLA) and leaf nitrogen content (LNC) are closely associated with photosynthesis and water retention (e.g., low SLA enhances WUE [17,18,19]). According to the leaf economics spectrum, plants exhibit a spatial trade-off between resource acquisition (high SLA, high N) and conservation (high tissue density). However, focusing solely on aboveground traits provides an incomplete picture. The root economics spectrum and foraging strategies, exemplified by specific root length (SRL) are pivotal for regulating soil nutrient and water uptake [20,21,22,23], especially in heterogeneous alpine soils. While community-weighted mean (CWM) traits can scale these physiologi cal mechanisms to ecosystem level, how aboveground and belowground traits coordinate (or diverge) to modulate the NUE–WUE relationship along environmental gradients remains an unresolved question.

The Changtang Plateau, characterized by a sharp precipitation gradient (50~700 mm) spanning arid in the west to semi-humid zones in the east, serves as an ideal natural laboratory to disentangle the intrinsic mechanisms linking plant strategies to ecosystem functions [24,25]. The growth season temperature remains relatively stable (spatial variance < 2 °C), minimizing temperature as a confounding factor in our analysis [26]. This natural climatic cline creates a continuous gradient in soil moisture and nutrient availability, with soil organic matter content decreasing from approximately 4.0% to below 1.0%, and soil total nitrogen (STN) content declining from 0.2% to around 0.02% [27]. Here, we integrate transect surveys with N-addition experiments to reveal how functional trait divergence regulates the spatial trade-off between RUE and NUE. Finally, it is crucial to distinguish between physiological trade-offs (e.g., between WUE and NUE at the individual or leaf scale) and spatial trade-offs (the contrasting spatial patterns of resource use efficiencies across environmental gradients). Our study focuses on the latter phenomenon, where RUE peaks in arid regions while NUE peaks in humid regions along the precipitation gradient, a pattern supported by coordinated functional trait divergence.

We hypothesize that: (1) As precipitation increases, the primary ecological filter shifts from hydraulic stress to nutrient competition; (2) This shift drives a community-level turnover from conservative species (high root investment) to acquisitive species (high leaf investment); and (3) This functional reorganization underpins a spatial decoupling of RUE and NUE, maximizing the limiting resource use efficiency at each end of the gradient. Our study addresses three critical gaps in the literature: (1) the community-scale validity of the spatial trade-off hypothesis across steep precipitation gradients; (2) the role of coordinated leaf–root trait divergence in modulating resource use efficiencies; and (3) the integration of field transect surveys with controlled N-addition experiments to establish causal relationships.

This study provides a functional ecological explanation for the adaptation mechanisms of alpine ecosystems and the geographical patterns of resource use. By explicitly distinguishing RUE from WUE, providing mechanistic context for precipitation-driven nitrogen dynamics, and clarifying the role of coordinated functional traits, this study advances our understanding of how alpine grasslands adapt to environmental gradients. Our findings offer a mechanistic basis for adaptive management in climate-sensitive alpine biomes and a scientific basis for formulating differentiated grassland management schemes to enhance ecosystem productivity.

Operational Definitions Summary:

RUE (Rain use efficiency): ANPP/MAP (g m^−2^ mm^−1^), community-scale precipitation use efficiency;

WUE (Water use efficiency): Physiological measure of carbon assimilation per unit water transpired (leaf level);

NUE (Nitrogen use efficiency): ANPP/soil available N (g g^−1^), community-scale nitrogen utilization efficiency;

FUE (Fertilizer use efficiency): ΔANPP/ΔN (g g^−1^), sensitivity of communities to exogenous nitrogen input.

## 2. Materials and Methods

### 2.1. Study Area

**Figure 1:**
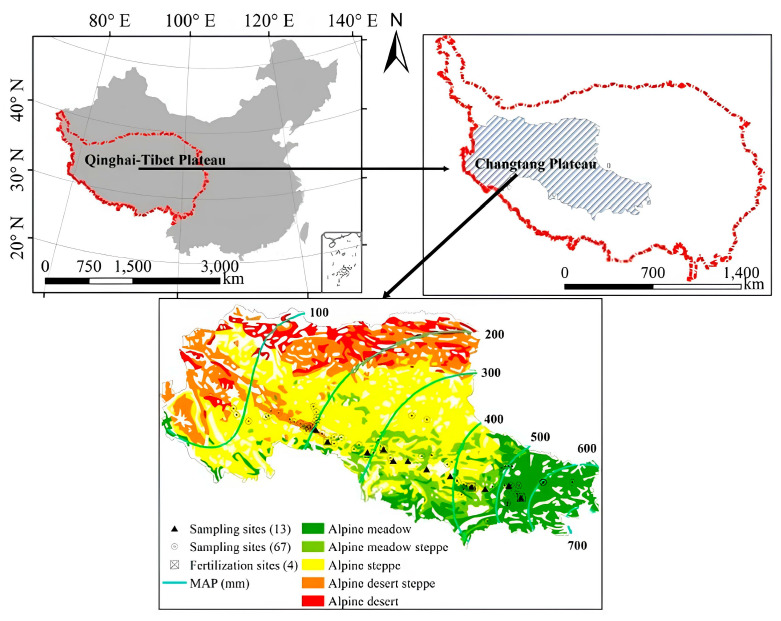
Distribution of grassland types and sampling sites on the Changtang Plateau.

The study was conducted on the Changtang Plateau, a vast alpine hinterland of the Tibetan Plateau (29°53′–36°32′ N; 78°41′–92°16′ E), with a mean elevation exceeding 4500 m. The region has a typical alpine arid climate, with mean annual aridity index ranging from 1.6 to 20 [28]. It is characterized by a distinct longitudinal precipitation gradient, where mean annual precipitation (MAP) declines sharply from ~700 mm in the east to <80 mm in the west (Figure 1), while growing season temperature remains relatively stable, with a spatial variance of <2 °C [29]. This natural climatic cline creates a continuous gradient in soil moisture and nutrient availability, with soil organic matter content decreasing from approximately 4.0% to below 1.0%, and soil total nitrogen (STN) content declining from 0.2% to around 0.02% [27]. The coupled water–nutrient gradient drives a sequential vegetation transition from alpine meadows (dominated by *Kobresia pygmaea*) in the humid east to alpine steppes (*Stipa purpurea* dominated) and desert steppes in the arid west. To elucidate the adaptive mechanisms of plant functional traits and resource use efficiency in response to water and nitrogen availability, we delineated the gradient into two functional zones based on the 400 mm isohyet: the semi-arid region (<400 mm) and the semi-humid region (>400 mm).

### 2.2. Data Acquisition and Analyses

The study adopted a combined approach of observational and experimental research to comprehensively analyze the spatial trade-off mechanism of functional traits differentiation and resource utilization efficiency in the northern alpine grassland of the Changtang Plateau. The observational research set up transects along the precipitation gradient of the northern Changtang Plateau (1200 km), systematically collecting data on vegetation, soil, and microclimate, covering a total of 67 sites; by integrating with Yang’s research data, this study set up 13 sampling points; the experimental research set up control and fertilization treatments at representative sites (4 sites) and conducted 3 rounds, observing the dynamic response of resource utilization efficiency. To ensure the comparability and integration of the two types of data, we established a unified analytical framework: (1) We standardized the calculation formulas for RUE and NUE in observational studies with those in experimental studies, ensuring consistent measurement standards; (2) In the integrated analysis, the precipitation gradient was used as an environmental background variable to control the influence of environmental heterogeneity on the two types of research results. Through this integrated analysis, we were able to distinguish the adaptive strategies under the natural environmental gradient (observational study) from the resource utilization responses under human intervention (experimental study), thereby more comprehensively revealing the ecological mechanism that drives the spatial trade-off of resource utilization efficiency through functional trait differentiation.

#### 2.2.1. Transect Survey and Biomass Sampling

During the peak growing season (July~August) of 2013~2016, we established a 1200 km transect spanning 67 sites along the precipitation gradient. At each site, five random 1.0 m × 1.0 m quadrats were systematically arranged (>500 m intervals) to minimize spatial autocorrelation. We quantified community structure (richness, coverage) and harvested aboveground biomass (AGB) by clipping at ground level. Belowground biomass (BGB) was determined using soil core (5 cm diameter, 0~30 cm depth) from three random points per quadrat. All plant samples were oven-dried at 65 °C to constant weight.

#### 2.2.2. Multi-Site Nitrogen Addition Experiment

To explicitly test the nutrient limitation and fertilizer response across the gradient, we established N-addition experiments at four sites representing distinct vegetation types (alpine meadow, meadow steppe, typical steppe, and alpine desert steppe). A randomized complete block design was employed with five N-addition levels (0, 2.5, 5, 10, and 20 g N m^−2^ yr^−1^) using urea, replicated five times (4 m × 4 m plots). A 2 m wide buffer zone was set between plots to prevent cross-contamination. Fertilizer was applied annually in late July to coincide with peak vegetative growth and maximize nitrogen absorption efficiency by plants. It is particularly important to note that the high-dose nitrogen addition experiments (such as 10 and 20 g N m^−2^ yr^−1^) are only applicable for response curve detection, but they do not align with production practices and the conservation goals of biodiversity in alpine grassland ecosystems.

#### 2.2.3. Soil Properties and Resource Use Efficiency

##### Soil Sampling and Soil Properties

Soil samples (0~20 cm) were analyzed for inorganic nitrogen (SIN), total nitrogen (STN), and organic carbon (SOC) using standard flow injection and elemental analysis protocols. Fresh soil samples were refrigerated and later extracted with 2 M/L KCl in the laboratory. Soil inorganic nitrogen (${NH}_{4}^{+}$, ${NO}_{3}^{-}$) content (SIN) was determined using a flow analyzer (BranLubbe AA3, manufactured by German Bran-Luebbe GmbH, and produced in Hamburg, Germany). The remaining soil samples were air-dried, ground, and sieved through a 100-mesh sieve. Soil organic carbon (SOC) content was measured using the potassium dichromate volumetric method, and soil total nitrogen (STN) content was determined using a C/N elemental analyzer (Elementar Vario Max, manufactured by Elementar Company from Germany, and produced in Hanau, Germany). Soil bulk density and soil moisture content were measured using the ring knife weighing method. Additionally, due to the high spatial heterogeneity of soil moisture content, soil organic carbon density, aboveground biomass, and underground biomass along the precipitation gradient, we supplemented our field data with publicly available datasets from Yang et al. (2008) [26]. Specifically, we incorporated the soil organic carbon density (SOC) and soil moisture content data from 122 sampling sites across the Tibetan grasslands, as well as the aboveground and belowground biomass estimates from 85 sites, all compiled in the *Global Change Biology* study by Yang et al. (2008) [26]. All data were standardized to the same units, and the interpolated values were used to fill gaps in our field measurements for sites with low sampling density. The supplementary data were integrated with our field measurements using a weighted average approach, where the weight of the supplementary data was determined by the spatial proximity to the field sampling site.

##### Calculation of Resource Use Efficiency

Rain use efficiency (RUE) is defined as the ratio of aboveground net primary productivity (ANPP) to mean annual precipitation (g m^−2^ mm^−1^), and is a commonly used indicator to characterize community-level WUE in grassland ecosystems [4,30]. However, RUE usually ignores the diversity of water sources and the key processes of water transmission and transformation within the soil–vegetation–atmosphere continuum (SPAC). Therefore, it cannot be fully equivalent or substitute for WUE. Nevertheless, in the specific hydrological and ecological context of the Qinghai–Tibetan Plateau, the water sources are relatively single (mainly precipitation), the hydrological processes are simplified (with uniform alpine meadows and grassland ecosystems), and the vegetation growth is synchronized with precipitation. RUE can overcome its inherent limitations to a certain extent, thus serving as a valuable and easily operable large-scale substitute indicator for WUE, which can be used to reflect the effective coupling relationship between community productivity and water. We use peak growing season AGB as ANPP in this study.

Nutrient use efficiencies were divided into two types of indicators to characterize nitrogen use efficiency, corresponding to responses at the community level under natural and fertilization conditions.

(1) Community-scale NUE: The ratio of ANPP to soil available nitrogen ($N_{available}$), It reflects the primary productivity that can be generated by the input of available soil nitrogen. It integrates soil processes (such as nitrogen mineralization and supply) and plant processes (such as plant absorption and transformation of nitrogen), thus cannot be completely equivalent to the nitrogen utilization efficiency within the plants. Especially influenced by the heterogeneity of the soil (uneven distribution of nutrients in space) and the dynamics of mineralization (which change dramatically over time), based on single-point, single-time soil sampling, there is uncertainty, which may mask the true plant nitrogen utilization efficiency. Furthermore, the nitrogen content used here is the area-basis stock, that is, g N m^−2^ yr^−1^. The soil samples were collected from the 0~20 cm soil layer, which is the main distribution layer of plant roots in the high-altitude grassland ecosystem of the Qinghai–Tibetan Plateau. The unit of NUE is obtained by the ratio of ANPP and the soil inorganic nitrogen content, that is, g g^−1^.

(2) Fertilizer use efficiency (FUE): The ratio of the increment in ANPP of fertilized plots (${ANPP}_{fertilized}$) relative to control plots (${ANPP}_{control}$) to the amount of nitrogen applied ($N_{addition}$, g N m^−2^ yr^−1^), reflecting the sensitivity of communities to exogenous nitrogen input.

#### 2.2.4. Plant Functional Traits and Community Scaling

**Figure 2:**
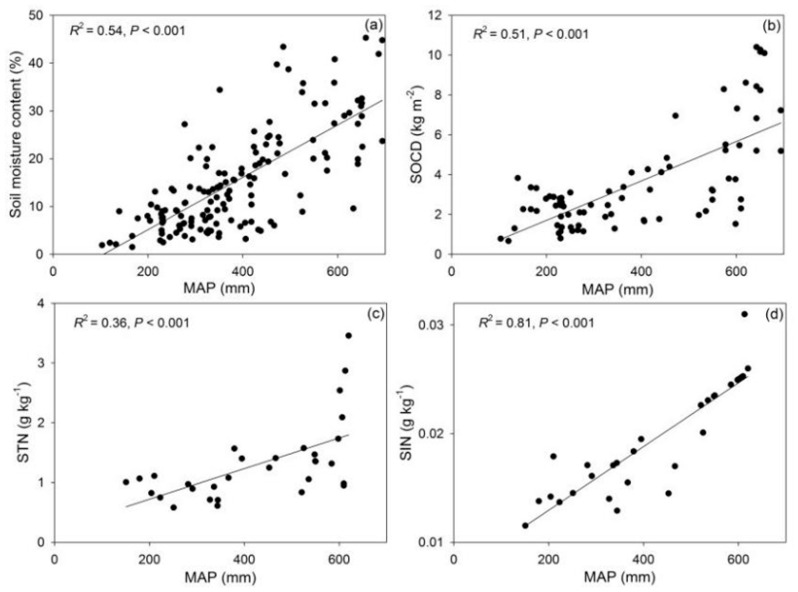
Co-variation in resource availability along the precipitation gradient, soil moisture content (**a**), soil organic carbon density (SOCD, (**b**)), soil total nitrogen (STN, (**c**)) and soil inorganic nitrogen content (SIN, (**d**)). SOCD: soil organic carbon density; STN: soil total nitrogen; SIN: soil inorganic nitrogen.

This study set up a total of 67 investigation sampling points in the alpine grassland area of the northern Qinghai–Tibetan Plateau. Due to the limitations in transportation and sample preservation, 13 sites were selected to collect samples of the leaf, root and soil of the dominant species of the grassland community during the peak period of the growing season (Table 1). These 13 sites were evenly distributed along the precipitation gradient, covering the main precipitation ranges from arid regions (50~150 mm) to semi-humid regions (400~600 mm) (Figure 2), and could represent the typical changes in the precipitation gradient in this area. To reduce the interference from terrain and grazing, all the sites were located in regional vegetation areas with flat terrain, far from human settlements, and with good plant growth. The adjacent sites were spaced 50~80 km apart to ensure the representativeness of the sampling points. At each site, species contributing 80%~90% of the total biomass were selected [31]. For each species, at least 20 healthy individuals were chosen to collect fully expanded green leaves, and at least five individuals were excavated to collect root samples (0~30 cm depth). Root samples were rinsed with deionized water to remove soil impurities. Three soil profiles (spacing ≥ 500 m) were randomly established at each site, and soil samples were collected from the 0~20 cm layer, where is the main root distribution layer of alpine plants. There may be uncertainties caused by insufficient characterization of plasticity.

We measured six key functional traits representing leaf and root economics spectra for dominant species (constituting > 80% of total biomass) [32,33,34]: (1) Resource acquisition and retention traits: specific leaf area (SLA) and specific root length (SRL); (2) Resource acquisition capacity and nutrient status traits: leaf nitrogen content per unit mass (${LN}_{mass}$) and root nitrogen content per unit mass (${RN}_{mass}$); (3) Gas exchange and resource transport traits: leaf nitrogen content per unit area (${LN}_{area}$) and root nitrogen content per unit length (${RN}_{length}$). Trait determination and calculation followed standardized protocols [31].

Leaf traits: Fresh leaves were scanned using a flatbed scanner (Epson Perfection V700, manufactured by Seiko Epson Corporation, and produced in Kofu City, Japan) to obtain total leaf area ($L_{area}$, cm^2^), then dried at 65 °C to constant weight to measure leaf dry mass ($L_{mass}$, mg)·(mg g^−1^). $L_{mass}$ was determined using an elemental analyzer (Elementar Vario Max, manufactured by Elementar Company from Germany, and produced in Hanau, Germany).

Root traits: Cleaned roots were scanned using a root image analysis system (WinRHIZO Pro, its download link is: https://www.regentinstruments.com/assets/winrhizo_software.html, accessed on 1 December 2025) to obtain total root length ($R_{length}$, cm), then dried at 65 °C to constant weight to measure root dry mass ($R_{mass}$, mg). ${RN}_{mass}$ (mg g^−1^) was determined using the same elemental analyzer.

Plant trait calculations are as follows:

(1) $$SLA=L_{area}/L_{mass}$$

(2) $$SRL=R_{length}/R_{mass}$$

(3) $${LN}_{area}={LN}_{mass}\times L_{mass}/L_{area}$$

(4) $${RN}_{length}={RN}_{mass}\times R_{mass}/R_{length}$$

where SLA is specific leaf area (cm^2^ g^−1^), SRL is specific root length (cm g^−1^), $L_{area}$ is the total leaf area from scanned images (cm^2^), $L_{mass}$ is the dry mass of scanned leaves (mg), $R_{length}$ is the total root length from scanned images (cm), $R_{mass}$ is the dry mass of scanned roots (mg), ${LN}_{area}$ is leaf nitrogen content per unit area (mg cm^−2^), ${LN}_{mass}$ is leaf nitrogen content per unit mass (mg g^−1^), ${RN}_{length}$ is root nitrogen content per unit length (mg cm^−1^), and ${RN}_{mass}$ is root nitrogen content per unit mass (mg g^−1^).

To scale these physiological traits to the ecosystem level, we calculated community-weighted mean (CWM) traits, with species relative biomass as the weight, integrating interspecific trait variations to reflect community-scale adaptation strategies.

(5) $$CWM=\sum_{i=1}^{n}p_{i}\times {trait}_{i}$$

where $p_{i}$ is the relative biomass abundance of species i and ${trait}_{i}$ is the trait value. This metric integrates interspecific turnover and intraspecific variability, serving as a proxy for community-level resource acquisition strategies.

### 2.3. Statistical Analysis

Data were tested for normality and homogeneity of variance prior to analysis. We used generalized additive models (GAMs) and linear regressions to quantify the responses of CWM traits and efficiencies (RUE, NUE) to the precipitation gradient. The spatial trade-off vs. synergy relationships were evaluated by analyzing the covariance between paired traits and efficiencies. All analyses were performed in R 4.2.0.

## 3. Results

### 3.1. Co-Variation in Resource Availability and Community Structure

**Figure 3:**
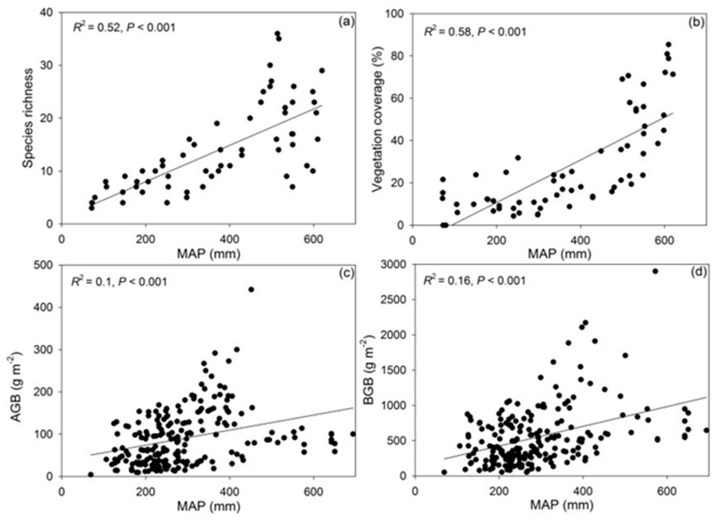
Plant community structure change along precipitation gradient, species richness (**a**), vegetation coverage (**b**), aboveground biomass (AGB, (**c**)) and belowground biomass (BGB, (**d**)).

Soil moisture, soil organic carbon density (SOCD), soil total nitrogen (STN), and soil inorganic nitrogen (SIN) all exhibited strong positive correlations (*p* < 0.001; Figure 2). This alleviation of resource constraints drove a concurrent expansion in community structure. Species richness, vegetation coverage, and biomass (both AGB and BGB) paralleled the environmental gradient, increasing significantly from the arid west to the mesic east (*p* < 0.001; Figure 3). Further statistical modeling confirmed that the interaction of water and nutrient availability explained the majority of variation in community assembly (Appendix A Table A1). Specifically, MAP, soil moisture and STN had significant impacts on grassland community structure (Figure 3).

### 3.2. Divergent Spatial Patterns of Resource Use Efficiency

**Figure 4:**
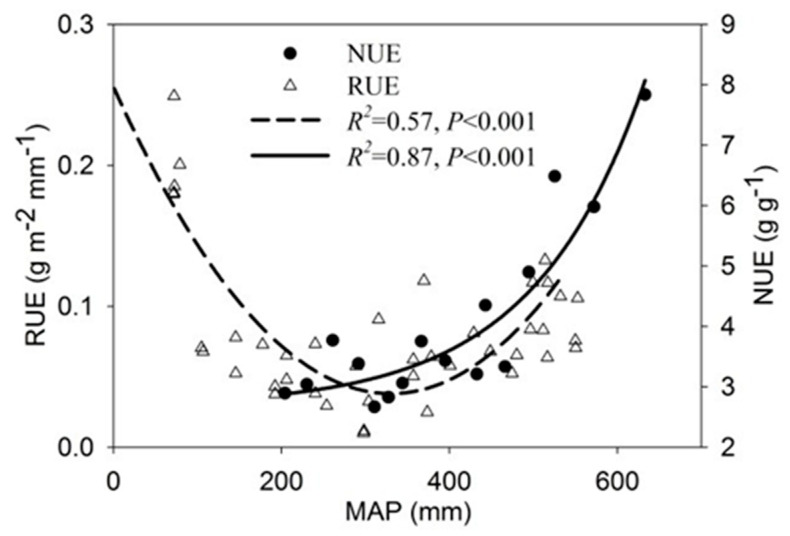
Spatial variation characteristics of rain use efficiency (RUE) and nitrogen use efficiency (NUE) in the precipitation gradient zone of the Changtang Plateau.

RUE followed a distinct quadratic function pattern (*p* < 0.001), peaking in the arid region (MAP < 400 mm) before declining in the mesic meadow (Figure 4). Community-scale NUE exhibited an exponential increase along the MAP gradient (*p* < 0.001), reaching its maximum in the high-precipitation eastern sector (Figure 4).

### 3.3. Intensification of Nitrogen Limitation in Humid Region

**Figure 5:**
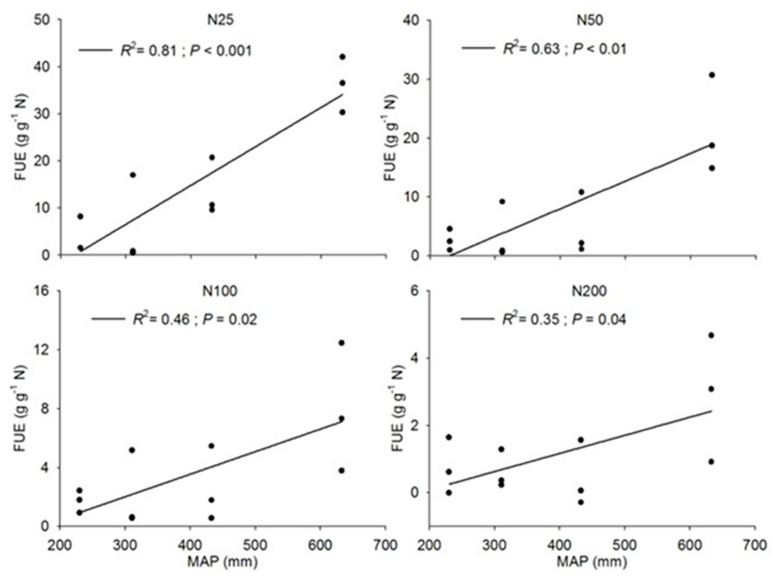
Fertilizer use efficiency (FUE) of different nitrogen application rates along precipitation gradient, with N25, N50, N100 and N200 representing nitrogen application rates of 2.5, 5.0, 10.0 and 20.0 g N m^−2^ yr^−1^, respectively.

Nitrogen addition experiments revealed a clear spatial pattern in nutrient limitation. FUE increased linearly with precipitation (Figure 5), indicating that grassland communities in humid regions are significantly more sensitive to exogenous nitrogen inputs than their arid counterparts. Furthermore, FUE exhibited diminishing marginal returns: the magnitude of growth response declined with increasing N dosage (Table 2), with the highest efficiency observed at low N addition rates (2.5 g N m^−2^ yr^−1^).

### 3.4. Shift in Functional Trait Syndromes

**Figure 6:**
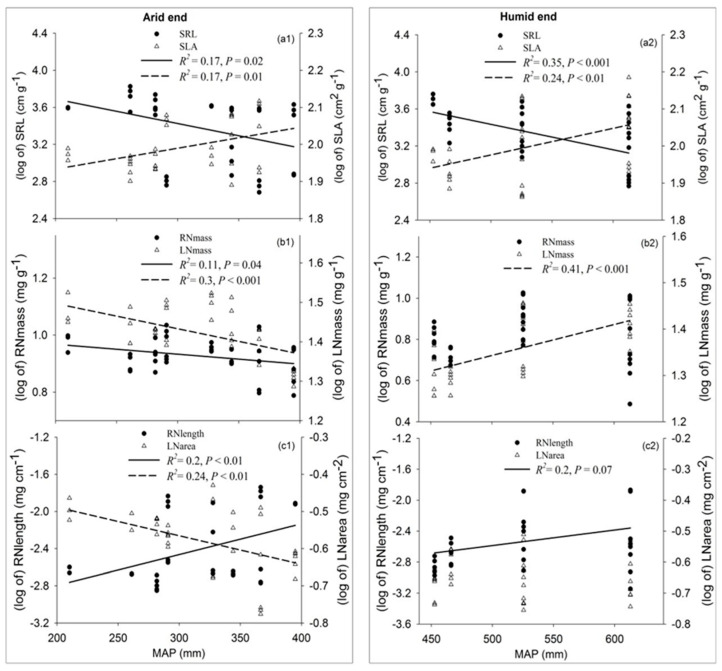
Spatial variation in paired traits on the aboveground and belowground in the precipitation gradient zone of the Changtang Plateau. SRL in arid end (**a1**); ${RN}_{mass}$ in arid end (**b1**); ${RN}_{length}$ in arid end (**c1**); SRL in humid end (**a2**); ${RN}_{mass}$ in humid end (**b2**); ${RN}_{length}$ in humid end (**c2**).

Community-weighted mean (CWM) traits revealed a coordinated shift in resource acquisition strategies (Figure 6). Along the precipitation gradient, vegetation transitioned from a belowground investment strategy in arid zones, characterized by high SRL, high ${LN}_{mass}$ and high ${LN}_{area}$ (Figure 6(a1,b1,c1)) to an aboveground acquisitive strategy in humid zones, characterized by high SLA and ${LN}_{mass}$ (Figure 6(a2,b2)). Specifically, as precipitation increased, plants traded root foraging capacity (declining SRL) for photosynthetic potential (increasing SLA). Interestingly, ${RN}_{mass}$ showed a stronger decline in arid regions compared to humid regions, reflecting distinct nutrient allocation priorities under different stress regimes.

## 4. Discussion

### 4.1. Shifting Limitations: From Hydraulic Stress to Nutrient Competition

The community-scale NUE of the precipitation gradient zone on the Qinghai–Tibetan Plateau increases with the increase in precipitation. The nitrogen addition experiment also shows that the FUE of grassland communities at each nitrogen application level increases continuously with the increase in precipitation. Grassland communities with high NUE have more obvious fertilization effects, greater productivity growth, and higher FUE. This indicates that the plant communities at the humid end have more nitrogen deficiency compared to those at the arid end. The grassland communities at the humid end have a more vulnerable response to nitrogen limitation in terms of biological productivity due to the moisture gradient. The response of productivity to the increase in precipitation is stronger than that of nitrogen mineralization to precipitation [35,36]. Although there is a strong positive correlation between productivity and the total amount of inorganic nitrogen in the soil [6], the response of productivity to the increase in precipitation is limited by nitrogen mineralization and available soil nitrogen, especially in the high-altitude environment of the Qinghai–Tibetan Plateau. The replenishment of available nitrogen may not be timely or sufficient to compensate for the nitrogen consumption caused by biomass formation [7,8]. Therefore, the humid area may be nitrogen-deficient due to a significant increase in productivity. To adapt to the nitrogen-deficient environment, the grassland ecosystems at the humid end may have more efficient nutrient utilization efficiency than those at the arid end. Our results demonstrate a critical threshold shift across the Changtang Plateau: when MAP exceeds 400 mm and alleviates hydraulic constraints, the dominant ecological filter transitions from water stress to nitrogen limitation. Thus, nutrient availability becomes the primary constraint in the more humid end of the gradient. Although soil nutrient status is generally poor across both arid and humid regions [37,38], the humid zone maintains a relatively higher soil N supply capacity. By contrast, the arid zone exhibits lower vegetation density, species richness, and ANPP, weakening interspecific nutrient competition for nutrients [39,40,41] while sustaining higher ${LN}_{mass}$ [42], such that alpine vegetation is unlikely to experience strong nutrient limitation.

Despite increasing soil total N along the precipitation gradient, the exponential rise in biomass creates a “dilution effect” that intensifies competition for available N [7,8,24,43]. This explains the observed paradox that humid communities, despite richer soil nutrients, exhibit higher FUE and stronger N limitation. With increasing MAP, vegetation density and species richness and ANPP begin to rise markedly [44,45], and intensified competition progressively strengths nutrient limitation [30,46]. Regional N addition experiments further confirm higher nitrogen use efficiency in the humid zone, consistent with more sever N limitation. Compared with the arid region, grassland communities in the Humid grassland communities therefore exhibit greater N demand and rely on efficient nutrient conservation and uptake strategies. Accordingly, vegetation in humid regions shifts toward an aboveground acquisitive strategy, prioritizing high SLA over root foraging (low SRL) to maximize light competition and carbon gain. Plants enhance photosynthetic capacity and nutrient use efficiency via coordinated trait adjustments: higher SLA and reduced SRL, with roots relying on increased surface area rather than extended foraging length to acquire nutrients.

Across the precipitation gradient, ${LN}_{mass}$ is higher in the arid region and declines with increasing MAP but rises sharply above 400 mm while ${RN}_{mass}$ remains unchanged. This indicates that as water limitation eases, plants reallocate N preferentially to aboveground tissues to boost photosynthesis and nutrient use efficiency [17,33,47,48]. The decoupling of stable ${RN}_{mass}$ and increasing ${LN}_{mass}$ reflects a strategic shift in limited N toward photosynthetic organs, optimizing NUE by reducing redundant root investment.

With increasing precipitation, SLA and ${LN}_{mass}$ increase continuously, whereas ${LN}_{area}$ is unchanged. Meanwhile, SRL decreases, ${RN}_{mass}$ remains stable, and ${RN}_{length}$ increases. This demonstrates that in the nutrient richer humid east, plants downregulate root foraging capacity and invest more in aboveground photosynthetic function, forming a clear trait spatial trade-off between enhanced aboveground productivity and conservative belowground resource acquisition.

### 4.2. Conservation Strategy Maximizes Rain Use Efficiency in Arid Zone

The core limiting factor in the arid zone of the Changtang Plateau is water. Our research has revealed a significant correlation between plant functional traits and ecosystem efficiency, but this correlation is not a simple direct causal relationship. Instead, it is achieved through specific ecological mechanisms. According to the TBP (trait-based productivity) theoretical framework, we distinguished two dimensions: quantitative traits (such as the total nitrogen content per unit area of leaves) and efficiency traits (such as the average nitrogen content of leaves). In the northern alpine grasslands of the Changtang Plateau, we observed that: in arid areas, the plant communities exhibited high efficiency traits and high RUE, which is consistent with the characteristic of higher efficiency traits in resource-poor regions; in humid areas, the plant communities showed high quantitative traits and high NUE, which is in line with the characteristic of higher quantitative traits in resource-rich regions. Specifically speaking:

First, plants morphologically prioritize water acquisition and retention. On the one hand, species increase SRL to maximize the root absorptive surface area for scavenging scarce soil moisture [17,37,49,50]. On the other hand, they significantly reduce SLA. This investment in leaf structure density is associated with reduced transpirational water loss [51,52,53] and may contribute to enhance leaf persistence under the stressful conditions common in open habitats [54,55,56].

Second, plants physiologically navigate the spatial trade-off between photosynthesis and transpiration through which stomatal closure prevents desiccation but restricts carbon uptake [5,10]. This study shows that plant communities in the arid zone have the highest RUE. To compensate for this limitation, arid species have higher ${LN}_{mass}$, maintaining a specific photosynthetic rate under low water and low CO_2_ concentrations [17,47,48,57]. This high nitrogen investment strategy under water stress may serve two functions: (1) increasing nitrogen allocation to non-photosynthetic organs or tissues, potentially improving water retention capacity [58,59]; and (2) resulting in higher ${LN}_{area}$, thereby enabling more efficient utilization of the region’s intense solar radiation [53,60] and helping to maintain internal CO_2_ gradients [61]. Meanwhile, maintaining lower ${RN}_{length}$ in the arid region may be beneficial for reducing root respiration rate or extend root lifespan [32,62].

Third, the resource allocation pattern reflects a slow-growing strategy and conservative life history [61]. Compared with leaf investment, plants tend to allocate a higher proportion of N to ${RN}_{mass}$. This acts as a resource bank, improving root retention and resource acquisition capacity [37], while providing guarantees for growth through root storage, thereby reducing dependence on immediate soil nutrient availability [63]. Concurrently, maintaining lower ${RN}_{length}$ helps reduce root respiratory costs or extending root lifespan [32,62].

### 4.3. Functional Trait Divergence Underpins the Spatial Decoupling of Efficiencies

Although relying solely on RUE and NUE to explain the physiological resource utilization efficiency of plants may have significant limitations, for instance, RUE often neglects the diversity of water sources for plants and the key processes of water transmission and transformation within the soil–vegetation–atmosphere continuum (SPAC); while NUE is not only affected by the internal nitrogen absorption and transformation of plants, but also greatly influenced by soil heterogeneity and the dynamic mineralization, RUE and NUE are more descriptive rather than physiological indicators, and usually cannot directly explain the carbon acquisition capacity and nitrogen utilization efficiency within plants. However, in the Changtang Plateau, precipitation is the main water source for ecosystems such as alpine meadows and alpine grasslands, and the hydrological process is relatively simple, with the growing season and the rainy season being synchronous. Therefore, RUE and NUE can be used as practical indicators for rapidly assessing the water and nutrient utilization functions of ecosystems on a large scale.

Integrating our findings, although RUE and NUE are often discussed in terms of physiological trade-offs at the individual or leaf level, our study reveals a different phenomenon at the ecosystem scale: a spatial decoupling of these efficiencies along the precipitation gradient. This spatial decoupling is not a direct negative correlation between RUE and NUE at any single site, but rather a contrasting spatial pattern where RUE peaks in arid regions while NUE peaks in humid regions. This pattern is mechanistically underpinned by the coordinated divergence of functional traits, with communities shifting from a water-conservative strategy (high SRL, low SLA) in arid regions to a nutrient-acquisitive strategy (high SLA, low SRL) in humid regions. This functional trait plasticity acts as a biological lever, allowing communities to shift along the resource economics spectrum. In the arid west, a conservative trait syndrome enhances resistance to hydraulic failure, whereas in the mesic east, an acquisitive trait syndrome helps overcomes nutrient dilution. This framework helps explain why a full synergy between water and nitrogen use efficiency is unlikely to be achieved across the steep climatic gradient of the Changtang Plateau.

Of course, in the future, if it is necessary to further verify and explain the resource utilization efficiency and strategies within plants at the mechanistic level, it will be necessary to directly measure the physiological WUE and NUE of plant communities through methods such as eddy covariance and isotopes.

### 4.4. Implications for Overcoming Vegetation and Biogeochemical Constraints

Validated by the distinct spatial trade-offs in resource use efficiencies, we propose a trait-based management framework that moves beyond one-size-fits-all policies to address the dynamic interplay between vegetation constraints (structural limitation) and biogeochemical constraints (nutrient limitation), based on the principle of adaptability in ecological management.

In the arid western steppe (<400 mm), ecosystem function is primarily limited by the sparse structure of the community itself rather than nutrient supply. Consequently, management must prioritize structure preservation. Suggested measures, such as grazing exclusion or rotational resting, should be implemented to minimize physical disturbance and protect the slow-growing, water-conservative functional types, e.g., high SRL and high RUE that are critical for preventing desertification.

Conversely, in the humid eastern meadow (>400 mm), productivity is constrained by biogeochemical limitations, specifically, the intensifying N limitation induced by rapid biomass dilution. Here, strategies should shift to constraint alleviation. In scenarios such as restoring degraded grassland or enhancing the productivity of artificial grasslands within a short period of time, we recommend low-dose nutrient amendment or the interseeding of N-fixing legumes to balance the soil N:P stoichiometry. This targeted approach relieves the physiological bottleneck, thereby maximizing the high production potential (high NUE) of the community without inducing eutrophication. However, in the long term, prolonged nitrogen addition may bring about ecological risks such as eutrophication, loss of biodiversity, and soil acidification. Therefore, management practices should adopt the principle of ‘prevention first’ to avoid irreversible damage to the ecosystem, while maintaining the flexibility of management strategies so as to make timely adjustments based on new ecological data and environmental changes.

Furthermore, while implementing the aforementioned management strategies, it is necessary to be vigilant about the long-term risks such as climate change that may affect the ecosystem of the Changtang Plateau. Studies have shown that the Qinghai–Tibetan Plateau is experiencing rapid climate change, which may lead to changes in precipitation patterns and thereby affect the current observed spatial decoupling pattern of RUE–NUE. Therefore, management planning should incorporate climate change scenario analysis, establish a risk warning mechanism, and promptly adjust management strategies.

### 4.5. Limitations and Future Directions

While this study provides insights into the shifting constraints along the precipitation gradient, several limitations should be acknowledged, pointing to valuable avenues for future research.

(1) How to effectively measure the photosynthetic rate and photosynthetic nutrient utilization efficiency of high-altitude plant leaves. Due to the small size and short height of the leaves of high-altitude plants (especially purple needle grass and Qinghai–Tibetan moss grass), it is difficult to measure the photosynthetic rate of the plants. In the future, seeking appropriate research methods and approaches for high-altitude vegetation will enable a more direct reflection of the relationship between plant traits and resource utilization efficiency, and explain the vegetation adaptation strategies at a deeper level of mechanism.
(2) Data on longer-term nitrogen fertilizer addition experiments need to be accumulated. The current nitrogen fertilizer addition experiments have obtained three-year data. Further research is needed on the response changes in grassland community structure and productivity after longer-term nitrogen fertilizer addition. The analysis of the impact of inter-annual precipitation differences on the nitrogen addition effect still requires the accumulation of data over a longer period. In particular, whether long-term nitrogen fertilizer addition will bring a series of changes in the aboveground and underground functional traits of vegetation and nutrient utilization strategies still requires further experimental verification.
(3) Conduct homogeneous garden (common garden) or interplanting experiments. The original plan was to conduct homogeneous garden or interplanting experiments, transplanting the main dominant species from different typical communities onto a common botanical garden under a gradient of precipitation. By setting gradients of water and nitrogen addition, the influence of environmental variation on the selection of functional traits and resource utilization strategies was analyzed, and the relationship between functional traits and resource utilization strategies was clarified. However, it was difficult to cultivate and overwinter the plants of *Stipa purpurea* and *Elymus mongolicus*, and the survival rate was very low. Due to the failure of two transplantations, the experiment was adjusted. In the future, whether it is possible to collect grass seeds under the precipitation gradient and conduct germination experiments to further verify the relative importance of key limiting factors in the precipitation gradient, whether there is a change pattern from the western water-limited area to the eastern nitrogen-limited area, and to reveal the synergistic relationship between plant functional traits and resource utilization strategies and their adaptation to environmental selection.

We must clearly state that this study is based on correlation analysis and correlation analysis can only indicate a statistical association between the two, cannot directly prove the causal relationship between functional trait differentiation and resource utilization efficiency. Therefore, we avoid using absolute expressions such as ‘prove’, ‘drive’, or ‘cause’, and instead use more cautious terms such as ‘correlation’, ‘support’, and ‘possibly reflect’.

## 5. Conclusions

Our study indicates that the spatial decoupling of water and nitrogen use efficiencies on the Changtang Plateau is associated with a coordinated divergence in plant functional traits. We identify a transition along the precipitation gradient, where ecosystem strategies shift from a water-conservative syndrome—characterized by traits like high specific root length and structural leaf properties that enhance RUE in arid steppes—toward a nutrient-acquisitive syndrome, marked by traits such as high specific leaf area that promote NUE in mesic meadows. This functional reorganization creates a functional spatial trade-off, a pattern further compounded by indications of nitrogen limitation in humid zones, as suggested by the observed trends in fertilizer use efficiency. Consequently, sustainable management could involve regionally adaptive approaches aligned with the following biological patterns: focusing on structural preservation to protect water-saving taxa in arid zones while considering moderate nutrient amendments in productive humid zones to alleviate potential constraints. This perspective provides a potential mechanistic basis for supporting ecosystem resilience under a fluctuating climate.

## Figures and Tables

**Table 1:** The location and environmental characteristics of sampling sites.

Site	Latitude/(°)	Longitude/(°)	Elevation/m	MAP/mm	MAT/°C	Dominant Species
1	31.2720	92.1495	4464	632.85	0.9	*Stipa purpurea*, *Carex moorcroftii*, *Potentilla bifurca*
2	31.5882	91.6590	4635	525.44	−0.4	*S. purpurea*, *C. moorcroftii*, *Saussurea tibetica*, *P. bifurca*
3	31.3971	90.8138	4619	466.13	0.1	*S. purpurea*
4	31.3942	90.3135	4632	432.63	0.2	*S. purpurea*, *C. moorcroftii*
5	31.6226	89.4819	4660	394.95	−0.7	*S. purpurea*, *P. bifurca*
6	31.7149	88.5858	4558	366.65	−1	*S. purpurea*, *S. tibetica*
7	31.8696	87.8611	4570	344.11	−1.4	*S. purpurea*, *P. bifurca*
8	31.7940	87.3316	4557	327.59	−0.9	*S. purpurea*, *C. moorcroftii*
9	32.0846	86.9078	4615	310.80	−1.5	*S. purpurea*, *P. bifurca*
10	31.9039	86.3425	4756	291.65	−0.8	*S. purpurea*, *P. bifurca*
11	31.9944	85.5666	4928	261.10	−0.6	*S. purpurea*, *C. moorcroftii*
12	31.9949	84.8298	4591	230.18	0.6	*S. purpurea*, *P. bifurca*
13	32.2682	84.3156	4498	204.25	0.7	*S. purpurea*

MAP: mean annual precipitation; MAT: mean annual temperature.

**Table 2:** Comparison of regression slopes of fertilizer use efficiency of different nitrogen application rates in the precipitation gradient zone of the Changtang Plateau.

Nitrogen Application Rate	n	$r^{2}$	p	$Slope\;\left(10^{-1}\right)$	Intercept	SlopeHeterogeneity (p)
N25	12	0.81	<0.001	0.92	−21.98	<0.001
N50	12	0.63	<0.01	0.59	−15.65	
N100	12	0.46	0.02	0.23	−5.49	
N200	12	0.35	0.04	0.09	−2.49	

## Data Availability

The dataset used in this article consists of three parts. The first part contains data from an academic research paper published by Yang in 2008. It includes soil organic carbon density and soil moisture data from 122 sampling points, as well as aboveground and belowground biomass estimates from 85 sampling points. The second part is data from 67 monitoring sampling points along major roads on the Changtang Plateau, with differences in precipitation gradients. The data mainly consist of plant traits data such as leaves and roots, and data on the utilization efficiency of water and nitrogen resources, which are observational data. The third part is experimental data on nitrogen element addition set up at 4 sampling points. The main data include comparative experiments on the absorption and utilization efficiency of different nitrogen element concentrations by vegetation. The data in the first part can be found in related research. The data in the second and third parts are from relevant institutions. Due to the regulations of the relevant departments, the original data cannot be published. Therefore, we are unable to provide the original data in accordance with the law. The data that can be displayed regarding the content of the paper have been presented in the article.

## Appendix A

**Table A1:** Pearson correlation coefficient between soil moisture, nutrient content and productivity indicators of grassland communities.

	Species Richness	Vegetation Coverage	Aboveground Biomass	Underground Biomass
Soil organic carbon	0.58 **	0.72 **	0.68 **	0.62 **
Soil total nitrogen	0.68 **	0.78 **	0.67 **	0.56 **
Soil inorganic nitrogen	0.61 **	0.72 **	0.8 **	0.7 **
Soil moisture content	0.65 **	0.66 **	0.56 **	0.27

“**” means that the result is statistically significant.
